# Infrastructure and Population of the OpenBiodiv Biodiversity Knowledge Graph

**DOI:** 10.3897/BDJ.9.e67671

**Published:** 2021-09-24

**Authors:** Mariya Dimitrova, Viktor E Senderov, Teodor Georgiev, Georgi Zhelezov, Lyubomir Penev

**Affiliations:** 1 Institute of Information and Communication Technologies, Bulgarian Academy of Sciences, Sofia, Bulgaria Institute of Information and Communication Technologies, Bulgarian Academy of Sciences Sofia Bulgaria; 2 Pensoft Publishers, Sofia, Bulgaria Pensoft Publishers Sofia Bulgaria; 3 Department of Bioinformatics and Genetics, Swedish Museum of Natural History, Stockholm, Sweden Department of Bioinformatics and Genetics, Swedish Museum of Natural History Stockholm Sweden; 4 Pensoft Publishers, Sofia, Bulgaria Pensoft Publishers Sofia Bulgaria; 5 Institute of Biodiversity & Ecosystem Research, Bulgarian Academy of Sciences, Sofia, Bulgaria Institute of Biodiversity & Ecosystem Research, Bulgarian Academy of Sciences Sofia Bulgaria

## Abstract

**Background:**

OpenBiodiv is a biodiversity knowledge graph containing a synthetic linked open dataset, OpenBiodiv-LOD, which combines knowledge extracted from academic literature with the taxonomic backbone used by the Global Biodiversity Information Facility. The linked open data is modelled according to the OpenBiodiv-O ontology integrating semantic resource types from recognised biodiversity and publishing ontologies with OpenBiodiv-O resource types, introduced to capture the semantics of resources not modelled before.

**New information:**

We introduce the new release of the OpenBiodiv-LOD attained through information extraction and modelling of additional biodiversity entities. It was achieved by further developments to OpenBiodiv-O, the data storage infrastructure and the workflow and accompanying R software packages used for transformation of academic literature into Resource Description Framework (RDF). We discuss how to utilise the LOD in biodiversity informatics and give examples by providing solutions to several competency questions. We investigate performance issues that arise due to the large amount of inferred statements in the graph and conclude that OWL-full inference is impractical for the project and that unnecessary inference should be avoided.

## Introduction

The OpenBiodiv system is a system uniting biodiversity knowledge extracted from academic publications and databases about biological diversity. It is based on a knowledge graph which aims to integrate knowledge sourced from articles from different journals and publishers and to allow querying of this knowledge through the establishment of semantic links within and between articles. Most recently, the general aspects of the system have been discussed and presented by Penev et al. (2019) and Dimitrova et al. (2019a). Previously Senderov and Penev (2016) introduced the software architecture of the system and Senderov et al. (2018) described the ontology OpenBiodiv-O, used for knowledge organisation. In the present paper, we build upon these works and elaborate on the most recent changes of the OpenBiodiv system: the programmatic approach used to create the linked open dataset OpenBiodiv-LOD and some of its applications. The most recent developments in OpenBiodiv provide direct solutions to some of the tasks recognised within the *Limitations and Future Directions* section of Penev et al. 2019, namely reuse of identifiers and enrichment of the knowledge graph with more resource types.

The existence of multiple biodiversity infrastructures which manage distinct datasets, (e.g. species occurrence data, taxonomic data, literature, sequence data etc.) has necessitated the establishment of a system to link these datasets (Sarkar 2007, Hobern et al. 2019). Using knowledge graphs for managing biodiversity knowledge has already been suggested in the biodiversity informatics community (Page 2016). In his conference presentation, R. Page outlines three different approaches towards constructing a biodiversity knowledge graph: 1) using crowd-sourcing (e.g. Wikidata), 2) from scratch, using predefined vocabularies and ontologies (e.g. OpenBiodiv, Ozymandias) and 3) via annotations linked to the associated evidence (Page 2020). The two biodiversity knowledge graphs OpenBiodiv and Ozymandias (Page 2019) both use a common approach and similar data sources (academic literature), but vary in the technical implementation and the vocabularies which are use.

We have chosen the knowledge graph technology as opposed to a relational database because it does not require a rigid schema from the beginning and allows us to add different entity types (e.g. RDF classes and properties) during different development stages of the project. We took full advantage of this by integrating additional resource types into OpenBiodiv, as described in more detail below.

The OpenBiodiv dataset comprises biodiversity information extracted from academic journals and public repositories of biodiversity data. OpenBiodiv-LOD is a synthetic RDF dataset, adhering to the Principles of Linked Open Data (Heath and Bizer 2011). It does not contain previously-unpublished data. Instead, it integrates information published by and extracted from academic journals and databases into a single dataset. However, some of the data present in OpenBiodiv-LOD have been logically inferred from statements in the original datasets and are, thus, novel. We propose to the biodiversity informatics community the use of OpenBiodiv-LOD as the central point for the biodiversity knowledge graph. The latest version of the OpenBiodiv-LOD is available in a GraphDB triple store (Ontotext 2020) under http://graph.openbiodiv.net, which provides a SPARQL endpoint for the repository OpenBiodiv2020 containing the dataset.

In the next sections, we discuss the sources of information that were combined to create the OpenBiodiv-LOD, the types of information that have been extracted, as well as the overall data model. We also discuss the Principles of Linked Open Data (LOD) that tie everything together. Finally, we discuss how the dataset was generated and demonstrate some of its applications using examples of SPARQL queries.

## Project description

### Title

OpenBiodiv

### Study area description

Biodiversity informatics and semantic publishing.

**The OpenBiodiv architecture**

The OpenBiodiv knowledge graph integrates biodiversity and publishing axioms contained in various ontologies, which combined form the OpenBiodiv-O ontology (Senderov et al. 2018), as well as statements with which this ontology is populated. Throughout this paper, we refer to these statements, or facts, as 'data', whereas all statements, describing the relationships between concepts, are understood as parts of an ontology.

**Figure 1.**
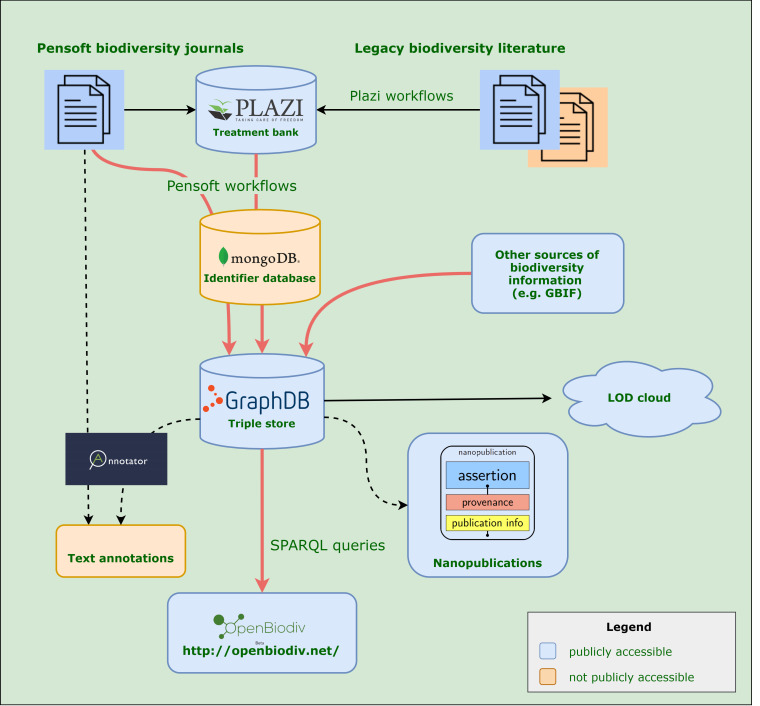
Information flows in the OpenBiodiv system. Red arrows show the workflows outlined in this paper. Two projects associated with the OpenBiodiv system are also shown: the Pensoft Annotator (Dimitrova et al. 2020) and a prototype workflow for generation of biodiversity nanopublications.

The data in OpenBiodiv-LOD comes from three major sources: from the GBIF Backbone Taxonomy (GBIF Secretariat 2019), from journal articles published by the academic publisher Pensoft and from Plazi Treatment Bank (http://plazi.org). These sources are illustrated in Fig. 1, which visualises the information flow in the OpenBiodiv ecosystem. In the next subsections, we describe each of these data providers in detail and the type of data that has been imported and integrated into OpenBiodiv-LOD.

**Data sources: the Global Biodiversity Information Facility (GBIF) backbone taxonomy**

GBIF is the largest international repository of occurrence data (GBIF Secretariat 2021). An occurrence record is a statement about the presence of an organism at a given place and time. GBIF allows its users to perform searches on its occurrence data by utilising a taxonomic hierarchy. For example, it is possible to query the database for mentions of organisms belonging to a specific genus - a search for mentions of taxa from the beetle genus *Harmonia* on 8 January 2021 returned 286 results (Fig. 2b). This search is possible thanks to the GBIF Backbone Taxonomy, also known as Nub (GBIF Secretariat 2019). Nub is a database organising taxonomic concepts into a hierarchy covering all biological names used in occurrence records harvested by GBIF. It is a single synthetic (algorithmically generated) management classification. Thus, the GBIF backbone does not represent an expert consensus on how biological taxa are hierarchically arranged according to evolutionary criteria in nature.

Keeping in mind this particular aspect of GBIF, it is evident how the backbone taxonomy allows GBIF to integrate name-based information from diverse sources of biodiversity information and to provide a facility for taxonomic searching and browsing. Some of the better known sources of information for GBIF include the Encyclopaedia of Life (EOL), GenBank and the International Union for Conservation of Nature (IUCN). In order to grant the same capabilities to OpenBiodiv-LOD, we have imported Nub as instances of openbiodiv:TaxonomicConcept according to the OpenBiodiv-O ontology (Senderov et al. 2018). A taxonomic concept is a biological name linked to an immutable circumscription as provided by an academic publication with the help of the keyword "sec." (Berendsohn 1995). Thus, each GBIF taxonomic concept is linked to an instance of openbiodiv:ScientificName and to a resource identifying a particular version of the GBIF backbone taxonomy. Furthermore, taxonomic concepts are linked to their parent taxonomic concept via a Simple Knowledge Organisation Schema (SKOS) (Miles and Bechhofer 2008) relation and via a fine-grained relation reified with the help of the Region Connection Calculus 5 (RCC-5) vocabulary that OpenBiodiv-O introduces (Senderov et al. 2018). These links constitute the taxonomic hierarchy in the case of SKOS and, in the case of RCC-5, the network of complex inter-relations between taxonomic concepts allowing overlaps and other special cases. A file containing the RCC-5 vocabulary used in OpenBiodiv (e.g.rcc5RelationTypes like ProperPart_INT) is available as a supplementary file in the OpenBiodiv-O ontology paper (Senderov et al. 2018).

The RCC-5 representation further allows the future evolution of OpenBiodiv-LOD to incorporate other simultaneous views of taxonomic alignment. For example, as the GBIF backbone taxonomy is updated regularly through an automated process from over 56 sources, future updates may be ingested as new statements into OpenBiodiv-LOD without altering existing records: namely, as a new set of taxonomic concepts and RCC-5 relations linked to potentially already-existing taxonomic names.

**Data sources: journal content from Pensoft and Plazi**

Pensoft is one of the leading publishers of journals on biodiversity. Its publications are open access and available as HTML, XML and PDF. Plazi is an aggregator specialising in harvesting of and providing access to legacy biodiversity publications openly on the web as XML. Articles from the journals listed in Table 1 have been converted to RDF and stored in the biodiversity knowledge graph OpenBiodiv. Additionally, taxonomic treatments from Plazi Treatment Bank, with the exception of those originally published by Pensoft, have been converted to RDF and stored in the graph as well. A taxonomic treatment is the special part of a taxonomic publication where the taxonomic concept circumscription (species description) takes place. The database is kept up-to-date with new publications on a rolling basis. The RDF-isation is made possible by the fact that all Pensoft journals are published as XML according to TaxPub, an extension of the NLM/NCBI journal publishing DTD for taxonomic descriptions (Catapano 2010) and, similarly, all Plazi treatments follow the TaxonX XML Schema (Penev et al. 2011). Thus, the RDF-isation pipeline does not require a natural language processing step, as a considerable amount of information is marked-up at the time of publication. An example of how a taxonomic name usage is marked up in the XML version of an article which follows the TaxPub schema is shown in Table 2.

The data types (article sections and other objects) which have been marked up in TaxPub and TaxonX, then converted to RDF and integrated in OpenBiodiv-LOD are listed in Table 3. Note that the marked-up data types do not correspond one-to-one to the RDF entities that have been created in the graph, as TaxPub, TaxonX and OpenBiodiv-O take slightly different approaches to modelling the biodiversity world. OpenBiodiv-O takes the most granular approach. For example, each taxonomic name usage in a Pensoft article results in a corresponding openbiodiv:TaxonomicNameUsage resource and a link to the openbiodiv:ScientificName resource that the taxonomic name usage mentions (Fig. 2a).

**Workflows and processes**

In this section, we explain how information from scholarly articles and the GBIF backbone is transformed into Linked Open Data which are stored and queried within the OpenBiodiv knowledge graph.

The inputs of the transformation pipeline are either XML (Pensoft and Plazi) or CSV (GBIF). Thus, the raw data-streams are semi-structured and the dataset generation problem can be thought of as an information retrieval and transformation problem. The input is encoded in three different data models: DarwinCore CSV (GBIF), TaxPub XML (Pensoft) and TaxonX XML (Plazi). The output of the transformation pipeline is knowledge represented in a fully-structured RDF according to the ontology OpenBiodiv-O.

**1. Obtaining the data**

GBIF’s taxonomic backbone is available at: https://www.gbif.org/dataset/d7dddbf4-2cf0-4f39-9b2a-bb099caae36c. There is an RSS feed from which Plazi treatments can be downloaded on a daily basis under http://tb.plazi.org/GgServer/xml.rss.xml. Each of Pensoft’s journals has a public API end-point under http://[journal_name].pensoft.net/lib/journal_archive.php?issue=xxx, where [journal_name] should to be replaced with the name of the Pensoft journal, for example, Zookeys to make https://zookeys.pensoft.net/lib/journal_archive.php?issue=1000. We use these sources of input to periodically obtain data and store it on our local servers.

**2. Tools**

In order to carry out the dataset generation, we made use of the following tools:

1. RDF4R and ROpenBio packages developed by us (https://github.com/pensoft/rdf4r, https://github.com/pensoft/ropenbio).
2. TSV4RDF, which is a PHP library for mapping CSV to RDF developed by us (https://github.com/pensoft/tsv4rdf) .
3. The OpenBiodiv base package (https://github.com/pensoft/OpenBiodiv).

In the rest of the section, we describe the transformation from XML as it is implemented in ROpenBio.

**3. XML to RDF transformation**

**Figure 3.**
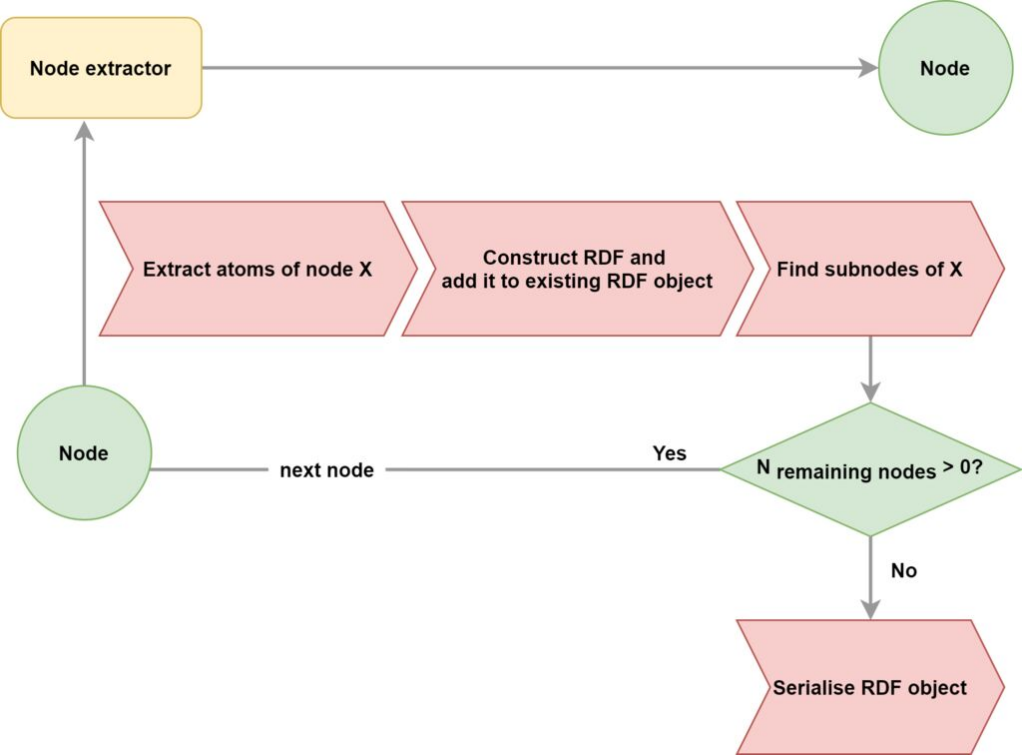
The Extractor procedure

In order to transform an article represented as an XML document to RDF, we make use of the hierarchical nature of XML and solve the problem recursively with the Extractor procedure, shown in Fig. 3. The procedure’s input is an XML node and its output is the RDF corresponding to the XML node. The extractor procedure has three essential steps: atoms extraction, RDF construction from the extracted atoms and a divide-and-conquer step that recursively calls itself on the sub-nodes and unites the results. Extraction of a whole article is achieved by calling the Extractor on the root node of the article.

**Information extraction from the article XMLs**

The atoms of an XML node consist of all text-fields that can be reached from the XML node with an XPATH expression (attribute values or text values) and can be directly converted to RDF as literals or identifiers. They all belong to one or to several related resources. For example, in Table 4 we have listed the XML node that contains author information in the TaxPub schema. The atoms here are surname = "Zhang", given_name = "Guanyang Zhang", orcid_id = "https://orcid.org/0000-0003-4389-4270", affiliation = "Florida Museum of Natural History, University of Florida, Gainesville, FL, United States of America"". In order to achieve the extraction, the atoms extractor must know the XPATH locations (e.g. the surname is at "./name/surname") of the authors it is looking for and the types of the values (e.g. string, integer, link etc.). Sometimes, this can be quite challenging as is the affiliation field in the given example. In it, the XPATH location of the address string depends on the value of xref. We were, however, unable to formulate a pure XPATH expression for the address string of a given author; in the production code, all addresses are extracted and additional logic in R matches the correct address. For example "//aff[id=./xref/@rid]" is the wrong idea: here "." no longer refers to the author object, but rather to the last matched object, i.e. the "aff" object.

**Divide-and-Conquer**

After atom extraction, we proceed to transform the content of each atom to RDF. This is done with a recursive call to the Extractor for all nodes that are hierarchically dependent on the current node. For example, the article node contains all the other other nodes such as sections, figures etc.

**Transformation specification**

In order for the Extractor to work, therefore, we need to specify an XML schema. The specification includes what XML nodes we are looking for and their location. It then recursively specifies for each node, what sub-nodes we are looking for and their XPATH location relative to their parent node. Finally, for every node, we need to give the atom locations and write a constructor. The transformation specification is done with the R6 framework in R. We have specified two schemata that share some constructors: one for TaxPub*^3^ and one for TaxonX*^4^.

In the most recent release of the OpenBiodiv-LOD and OpenBiodiv-O, we have introduced new resource types to represent biodiversity knowledge from the Materials Examined section and other elements of the article, such as links to the external genomic databases BOLD and GenBank, as well as the ORCID database for researchers. These changes to the ontology were also reflected in the TaxPub and TaxonX schema objects in the ROpenBio package, as well as the respective constructors which generate the triples from information extracted these schemas.

**RDF generation**

The process of RDF generation has three parts: (1) setting unique identifiers for each resource, (2) ascribing semantic classes to each resource and (3) linking resources via RDF properties.

Setting identifiers is an essential step to ensure that each resource can be uniquely identified across Linked Open Data. We use a MongoDB database (MongoDB, Inc 2021) to store and look-up resource identifiers and their associated labels as key-value pairs (e.g. key = OpenBiodiv identifier of article; label = article title), along with additional metadata, such as DOI. Identifier look-ups are performed via the function *get_or_set_mongoid* from ROpenBio, which performs a MongoDB query for a given resource label, depending on its resource type (i.e. article, treatment, author etc). To optimise text searching within our MongoDB database, we use sha256 hashing of the combination between the resource type and its label; the exact format of the combination is *type:label*. Thus, we check for a single word (the hash string), instead of the type and the label which most often contains multiple words and even paragraphs. Hashing is performed by the *set_values_to_sha256* function which makes use of the *sha256* function from the openssl package (Ooms 2020).

The *get_or_set_mongoid* function retrieves the identifier associated with the matched hash, so it can be re-used in semantic relations within the current RDF serialisation. If there is no matching record within the MongoDB database, the function generates a new universally-unique identifier using the *UUIDgenerate* function from the R package uuid (Urbanek and Ts'o 2020). We have found that obtaining identifiers using MongoDB lookups is faster compared to performing SPARQL queries to the GraphDB repository. The latter requires first a HTTP connection to the GraphDB server and then a query response from the graph database. Our comparison between the two methods found that MongoDB lookups are at least nine times faster (Suppl. material 1). This difference adds up in XML files with hundreds or sometimes thousands of extracted nodes, resulting in smaller processing times. In addition, the MongoDB database serves as a backup solution for all minted identifiers.

Organising resources into semantic classes, according to the OpenBiodiv-O ontology and creating links between them, is conceptually straightforward. For each atom, we know its type because the XML schema used to extract it contains a type field. Each type of resource has its own constructor function which generates RDF statements defining the resource types using rdf:type and links between resources. The author example is given in Table 4.

It should be noted here that the semantics of certain node types, such as taxonomic name usage (reified as :TaxonomicNameUsage), reflect the relative position of the node in the XML document. For example, a taxonomic name usage may be inside a figure, inside an introduction section, inside a title etc. Therefore, besides the atoms, the constructor receives information about the relative position of the resource in the article by means of the unique identifier of the parent node(s). Then this information is encoded in RDF as given in Table 5.

**4. Submission to graph database and post-processing**

The generated RDF statements are submitted to a repository in a GraphDB instance residing on http://graph.openbiodiv.net/. The repository, OpenBiodiv2020, has been initialised with OpenBiodiv-O*^1^, which links OpenBiodiv-O resources to resources from external ontologies*^2^. Finally, after the data has been submitted, update scripts are run to generate further statements for the updating of scientific name relations.

**Update rule for replacement name**. We state that a scientific name A replaces a scientific name B, if there exists a taxonomic name usage of A with taxonomic status :ReplacementName and B is mentioned by a taxonomic name usage in the nomenclatural citations of the treatment, where the discussed taxonomic name usage of A is in the nomenclature section (Table 6).

**Update rule for related name.** The related names update rule is similar to the one for a replacement name: two scientific names A and B are considered related if they are both mentioned in the nomenclature section of a treatment (Table 7).

For example, the names Muscidae and Aethiopomyia Malloch, 1921 are considered related (Table 8), an observation explained by the taxonomic relationship between them, as Aethiopomyia is a genus in the family Muscidae.

## Web location (URIs)

Homepage: https://github.com/pensoft/OpenBiodiv

Download page: 10.5281/zenodo.5283207

## Usage licence

### Usage licence

Creative Commons Public Domain Waiver (CC-Zero)

## Additional information

### Example SPARQL queries

We shall illustrate and evaluate the LOD by issuing sample SPARQL queries illuminating aspects of it.

**1. Simple queries**

**Query for author.** Authors are instances of foaf:Person (except in the rare institutional case, in which they would be foaf:Agent). The SPARQL query in Table 9 answers the question of which persons have been the most prolific authors in the harvested journals.

**Query for a scientific name.** Biological Latin names are stored in the system as :ScientificName and are mentioned by taxonomic name usages. Table 10 orders scientific names of any rank by the number of unique mentions that they have in articles. It is possible to narrow down the solution to binomial names (species names) by adding the dwc:specificEpithet and dwc:genus properties as shown in Table 11. It is also possible, for example, to determine the most-mentioned scientific name by the number of articles it is mentioned in (Table 12).

**Query the article structure.** A unique feature of OpenBiodiv-LOD is that articles are broken down to their components (see Table 3) and taxonomic name usages are connected to the specific part of the article and not just to the article in general. Combining this feature with queries from the previous paragraph, we can, for example, look for the most mentioned scientific name in a figure (Table 13) or for the figures present in a particular article (Table 14).

**Query for taxonomic concepts.** We can create a query uniting information from the GBIF Backbone Taxonomy with semantics coming from the article structure. The query in Table 15 locates taxa that are in the beetle family Curculionidae according to the taxonomic backbone of GBIF and looks for new taxa (:TaxonomicDiscovery) that have been associated with one of its genera.

**Fuzzy Queries via Lucene.** The SPARQL endpoint of OpenBiodiv-LOD supports fuzzy matching via a Lucene connector (The Apache Software Foundation 2013). This can be a very useful as, due to multiplicity of taxonomic names and the complexities of Latin grammar, one often does not remember the correct spelling of a name. The Lucene query needs to follow the standard Lucene query syntax (The Apache Software Foundation 2013) and is specified as a literal string of the property <http://www.ontotext.com/connectors/lucene#query> of the search variable (Table 16).

**Competency question answering via SPARQL**

**Validity of a name.** Of central importance to biological nomenclature is the question of whether a given taxonomic name is valid or not. We shall consider a taxonomic name invalid if and only if at least one of the following invalidation criteria holds:

1. The name has been replaced: i.e. there is a :replacementName property originating in the name and there are no loops (it is impossible to follow the :replacementName edges and come back to the name). This query is illustrated in Table 17.
2. The name has been invalidated: i.e. there is a taxonomic usage of the name with the status :UnavailableName and there is no newer taxonomic name usage revalidating it (:AvailableName). Illustrated in Table 18.

**The case of Museu Nacional de Rio de Janeiro (MNRJ).** In order to illustrate the capabilities of OpenBiodiv and draw attention to the scientific impact of the tragically lost collection in the fire of the Museu Nacional de Rio de Janeiro (MNRJ), we can ask our system to give us the number of times a specimen from that collection was used in a taxonomic article and in which ones (Table 19). We use MNRJ’s GRSCICOLL (GBIF Registry of Scientific Collections) identifier in addition to its name and collection code.

It turns out that MNRJ has been mentioned 362,062 times in our system in a total of 509 articles. Perhaps more interestingly, we can see specimens of which taxa may have been lost, have declining populations or are threatened by extinction. Examples include the insects (Xestoblatta, Charinus, Lamproclasiopa etc.) which are extinct, Keays's Rice Rats (*Nephelomyskeaysi*) which have declining populations and many others for a total of 1,348 distinct names mentioned in taxonomic articles which reference MNRJ.

**Specimen collection.** Some of the most important information about biodiversity in an article is within the Materials Examined section. It contains information about the collection of biodiversity samples (specimens), the location where they were found, the taxonomists who identified them, their habitats, the institutions where the specimens are kept and much more. For example, we can query all people who have collected specimens belonging to the insect genus *Zelus* (Table 20).

**Institutional impact.** We can use a SPARQL query to understand how collections from different instutions are used to describe taxa. In the example query, we have linked institutional identifiers with treatments which mention them to find out institutional impact per family (Table 21).

**Links between holotype descriptions (literature), institutions holding the holotypes and genomics.** OpenBiodiv integrates information about examined specimens and taxonomic descriptions from literature with external identifiers of institutions holding the specimens, as well as records identifiers pertaining to the genomic sequences of the specimens. We can retrieve this information with the SPARQL query in Table 22.

### Discussion

**Fulfilment of the Principles of Linked Open Data**

Linked Open Data (Heath and Bizer 2011) is an idea of the Semantic Web (Berners-Lee et al. 2001) aimed at ensuring that data published on the Web are reusable, discoverable and, most importantly, that pieces of data published by different entities can work together. The principles of LOD are the following (Heath and Bizer 2011):

1. Use URIs as names for things.
2. Use HTTP URIs so people can look up these things.
3. When someone looks up a URI, provide useful information, using the standards (RDF, SPARQL).
4. Include links to other URIs so they can discover more things.

We have followed these guidelines when creating the OpenBiodiv-LOD. We will now discuss each of these points separately.

**Usage of URIs as resource identifiers.** Every instance in OpenBiodiv-LOD is uniquely identifiable by a HTTP URI of the following form: http://openbiodiv.net/uuid-(suffix). All instance identifiers in OpenBiodiv LOD follow this schema. The optional suffix field is assigned only to resources extracted from GBIF.

**Identifiers for resources from Pensoft and Plazi.** During the RDF-isation of the sources Pensoft and Plazi, when a new concept is discovered (e.g. a person, a scientific name etc.), a UUID is generated. Then the resource is always referred to in the database by this UUID in the OpenBiodiv namespace, http://openbiodiv.net/. Pensoft and Plazi furthermore share the UUID part of the identifier in the semi-structured representation of treatments. For example, Lyubomir Penev is a resource identified by http://openbiodiv.net/416FDF84-1029-4115-B43F-E9E734004489.

**Identifiers for GBIF taxonomic concepts.** GBIF offers its taxonomic backbone as a DarwinCore (Wieczorek et al. 2012) tab separated file (TSV). Each row in the TSV corresponds to a taxonomic concept published by GBIF. GBIF does not offer a globally-unique ID of its concepts, but only a local ID (e.g. 4239 is the GBIF ID for their concept for weevils, Curculionidae sec (GBIF Secretariat 2019). This is why we generated a UUID (here: F1DD0CF0-217D-422B-BAA4-58901976D7B4-4239) for each row of the data table published from GBIF. Each taxonomic concept is linked to a taxonomic name and to a taxonomic concept label (name + “sec." + reference to the literature). It was impractical for programmatic reasons to generate a new UUID for these linked entities. This is why their unique identifiers are suffixed. We use the suffix -scName to denote scientific names and -label to denote taxonomic concept labels (e.g. http://openbiodiv.net/F1DD0CF0-217D-422B-BAA4-58901976D7B4-4239-label)

**Usage of HTTP URIs and dereferencing.** As per the Linked Data principles, we use dereferenceable HTTP URIs for our resources. For example, if a web browser opens http://openbiodiv.net/416FDF84-1029-4115-B43F-E9E734004489, a webpage is displayed (Fig. 4) providing useful information for the person Lyubomir Penev, such as his affiliations, research output and co-authors. In addition, it is possible to request OpenBiodiv resources via Curl with the header Content-Type application/rdf+xml and an RDF representation of the resources is returned.

**Linking to other resources.** All resources in OpenBiodiv form a graph (there are no disconnected parts), following a data model discussed in the next subsection. Second, resources are linked to external databases via properties like datacite:hasIdentifier and openbiodiv:mentionsIdentifier. These identifiers can be: GBIF IDs, ZooBank IDs, Zenodo IDs, ORCIDs, BOLD BINs, BOLD Records or GenBank accession numbers. We have created links between people and their ORCID records, publications and their GBIF dataset records, as well as Zoobank records and genomic records within BOLD and GenBank. See Table 21 for a SPARQL query which demonstrates the linking of taxonomic name data with external institutional identifiers from the GBIF Registry of Scientific Collections (GRSciColl) and Table 22 for a query which retrieves taxonomic circumscriptions of all holotypes, which have genomic identifier(s) and associated institutional records, which signify where the examined materials are stored.

**Data Model**

**Figure 5.**
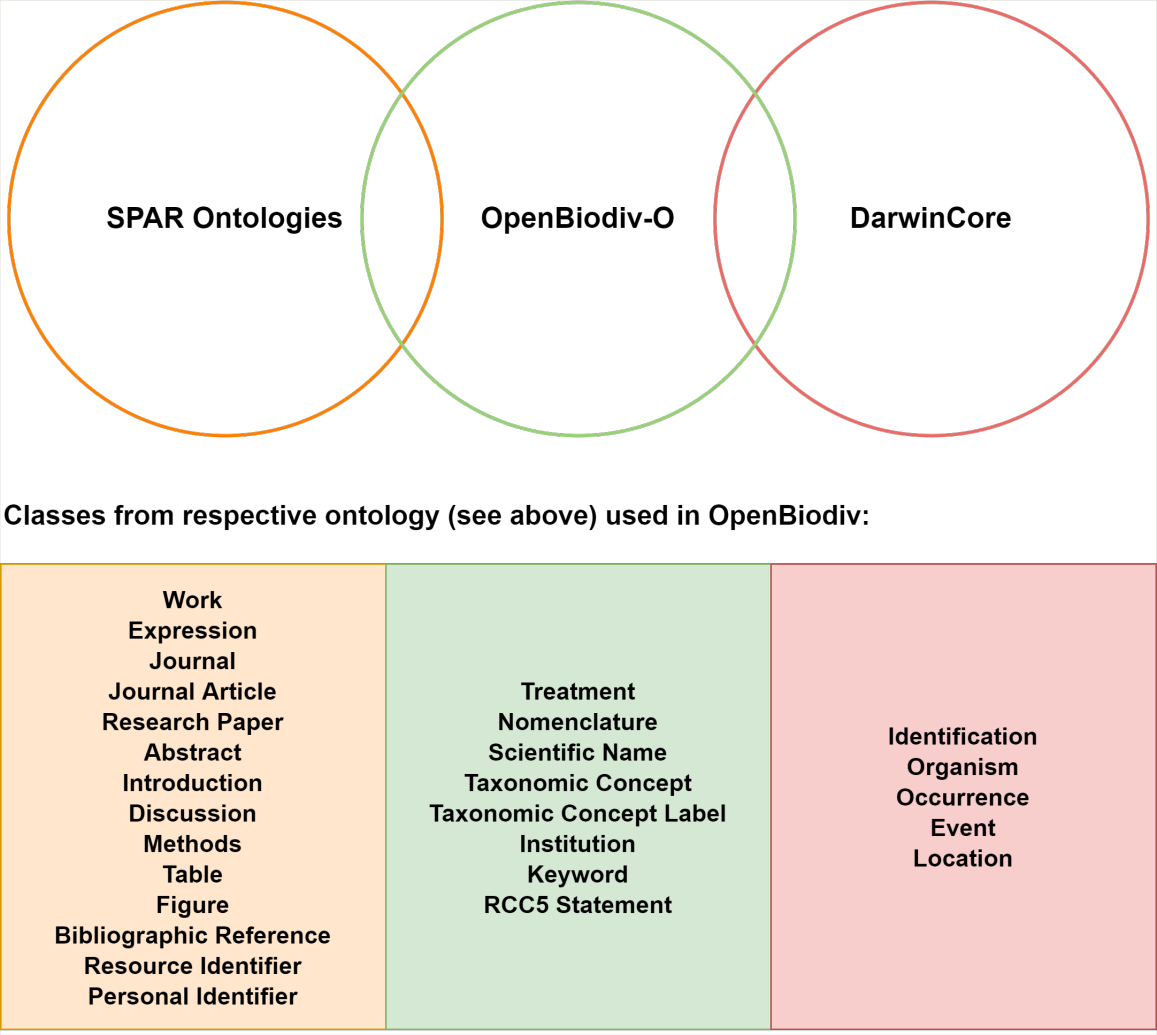
OpenBiodiv-O is an ontology that links the publishing domain with the biodiversity domain. Major resource types covered by each of the ontology families are given in the box below the Venn diagram. Each of them is present in the OpenBiodiv-O ontology as a class. Important resources from the publishing domain are listed in the left-most column and from biodiversity informatics in the right-most column. The middle one covers important OpenBiodiv-O resources.

When creating the RDF graph, we have conformed to the OpenBiodiv-O ontology described in Senderov et al. 2018, Dimitrova et al. 2019b and well-established community ontologies (Fig. 5). In particular, (1) we use the Semantic Publishing and Referencing Ontologies (SPAR, (Peroni and Shotton 2018) to model entities from published documents such as Journal, Article, Section, Figure, Table and so on; and (2) we use the DarwinCore (DwC) (Wieczorek et al. 2012) community standard to model entities in the biodiversity domain.

SPAR provides facilities to deal with the dichotomy between the abstract representation of knowledge through the class Work and its concrete representation through the class Expression. For example, a fabio:JournalArticle can be the realisation of a fabio:ResearchPaper. On the other hand, the DwC community standard gives a standard way to express properties from taxonomy and biodiversity science.

In the most recent verision of OpenBiodiv-LOD (Dimitrova et al. 2019b), we have represented occurrences, taxonomic identifications, locations and events via the respective DwC classes dwc:Occurrence, dwc:Identification, dcterms:Location and dwc:Event, as well as various other DwC properties which model information related to these four resource types (e.g. coordinates, type statuses, life stages). We have also integrated classes and properties from the DataCite ontology, part of the SPAR ontologies (Peroni and Shotton 2018), to model external resources via their identifiers. In particular, we have implemented the class datacite:ResourceIdentifier to model Zoobank, Zenodo, BOLD and GenBank identifiers and datacite:PersonalIdentifier to model ORCIDs. The property datacite:hasIdentifier has also been implemented to help establish links between existing resources and their external identifiers.

**Performance**

The current iteration of the database holds over 360 Mln triples. The expansion ratio under the RDFS-Plus (Optimised) ruleset is 1.20, i.e. for each asserted statement, we materialise, on average, 1.20 implicit statements. In a previous release of the dataset, which was under the OWL2-RL rule-set, the most complex rule-set supported by GraphDB, the expansion ratio was about 3.7; however, we encountered significant performance issues using it. Even with the lighter rule-set (RDFS-Plus Optimised) (Ontotext 2021), we still see performance degradation with increasing database size.

We observed a steady increase of implicit (inferred) statements during the upload of new triples. An example of such an inferred statement is :A po:contains :B, generated from the statement :*B po:isContainedBy :A* because po:isContainedBy is an inverse property of po:contains. Upon closer inspection, it turned out that the import of external ontologies, in addition to OpenBiodiv-O, leads to the generation of superfluous inferred statements. For instance, in the SKOS ontology, the property skos:exactMatch is transitive and is also a sub-property of skos:closeMatch. The same ontology defines that skos:closeMatch is a sub-property of skos:mappingRelation. Therefore, after importing the SKOS ontology, GraphDB infers that all treatments which have the property skos:exactMatch (these are only Plazi treatments for which we have information about their Plazi treatment id, for example, *openbiodiv:03894A65-5824-FFE9-571B-B65D2F47F95E skos:exactMatch plazi_treatment:03894A65-5824-FFE9-571B-B65D2F47F95E*), also have an additional statement with property skos:mappingRelation. This inferred statement does not actually bring any new semantic information to the knowledge graph, hence we consider it superfluous.

We came to the conclusion that all necessary RDF logic is stored in OpenBiodiv-O and does not require the import of other ontologies, since OpenBiodiv-O already includes the essential relations from these ontologies. Therefore, in the latest release of the repository, we have only imported the OpenBiodiv-O ontology.

Another important aspect of performance is the RDF-isation time or the time it takes to convert a single XML into RDF in trig serialisation and to upload it to the database. Our observations show that the most time-consuming part of this process are the MongoDB requests used to get and set resource identifiers. Even though they provided an improvement to the previous model, which used queries to GraphDB to obtain and set identifiers, MongoDB requests can add up to a significant amount of time per XML document. We noted that adding a MongoDB index and using it to search for text content does not improve the speed at all. As an alternative solution, we now use sha256 hashing to compact value strings associated with identifiers to a fixed-length hash string. This method is explained in detail in the Methods section.

### Conclusion

The generated dataset OpenBiodiv-LOD, similar to the expanded ontology OpenBiodiv-O, is already a solid resource for biologists, as it includes information from most articles published by Pensoft and Plazi and counts over 360 million RDF triples.

An important conclusion that can be drawn from the work is that it is possible to use a semantic graph for the integration of a large volume of data on biodiversity. We were unexpectedly given the opportunity to illustrate the power of the knowledge graph by analysing the damage from the tragic fire at the Museu Nacional in Rio de Janeiro. In addition, we have illustrated that it is possible to write relatively simple logical conclusions to check the validity of a taxonomic name.

Due to the large amount of data, we found that, although the use of a semantic graph was possible, some of the initially-chosen technologies proved to be inapplicable or difficult to apply. We have observed that the practical application of the full logical OWL model is difficult due to performance problems. Instead in the end, we utilised RDFs which are less powerful, but faster. In addition, we found that triple stores are not a universal solution to all data integration problems, but can be used in combination with other database technologies (e.g. MongoDB) to efficiently store and query semantic resources.

A great difficulty was the disambiguation of resources, such as author names or taxonomic names. In the functional design of the RDF4R package, we have input modules that allow us to insert a list of functions/rules for disambiguation when searching for an identifier for a given resource. However, we had only limited success with the rule-based disambiguation and, for this reason in the production system, it was discontinued for the moment.

Considering these and other "lessons", the future development of the OpenBiodiv-LOD project can be outlined in the following way:

1. As an immediate goal, to optimise the workflow for processing XMLs. This would be achieved by distributing the work across multiple workers operating on different machines.
2. Improve the search functionalities of the OpenBiodiv website to enable more user-friendly querying of the knowledge graph without requiring any undertanding of SPARQL from the users. We have identified user groups (biologists, taxonomists, institutions etc.) and respective competency questions which the website would aim to answer via different "apps". These apps will provide an interface in which users will fill in text fields and use a simplified faceted search functionality to answer different biodiversity questions and narrow down their answers. We could potentially augment the search functionality of the OpenBiodiv website by implementing additional semantic entities, extracted from articles via the Pensoft Annotator (Dimitrova et al. 2020), a text-to-ontology mapping tool developed by Pensoft.
3. Integrate OpenBiodiv with a nanopublication publishing service. Nanopublications (Groth et al. 2010) are a type of publication aiming to store and attribute even the smallest bit of information which can be expressed as a semantic triple. They follow certain guidelines (Gray et al. 2020) defining their constituent graphs (Assertion, Provenance, Publication Info) and the links between them. OpenBiodiv is a potential source for nanopublications since it already contains semantic statements about biodiversity (assertions), as well as provenance information, encapsulated in the publication metadata. As such, it can be used to generate nanopublications which could become part of the existing decentralised network of nanopublications (Kuhn et al. 2016). Existing nanopublication infrastructure and publishing formats can also help to establish a system for commenting, supporting and contradicting biodiversity statements extracted from literature.

We envision OpenBiodiv-LOD as an integral part of the existing semantic network of biodiversity knowledge, based on HTTP identifiers and controlled vocabularies. By semantically enhancing and linking knowlege in OpenBiodiv to existing machine-readable data, we augmment biodiversity data quality and increase the potential for its reuse.

## Supplementary Material

91052D25-AA61-59A4-BC4C-D5CD25CC9757 10.3897/BDJ.9.e67671.suppl1 Supplementary material 1 Comparison between GraphDB identifier look-ups and local MongoDB identifier look-ups Data type R script File: oo_529223.r https://binary.pensoft.net/file/529223 Mariya Dimitrova

## Figures and Tables

**Figure 2a.**
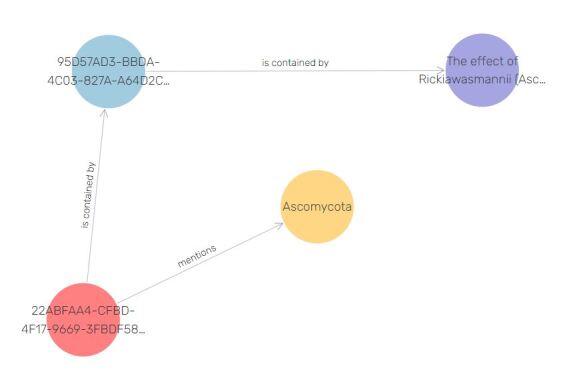
A taxonomic name usage (http://openbiodiv.net/22ABFAA4-CFBD-4F17-9669-3FBDF5897892) is linked to the scientific name it mentions, Ascomycota and to the part of the article (abstract) it is contained in.

**Figure 2b.**
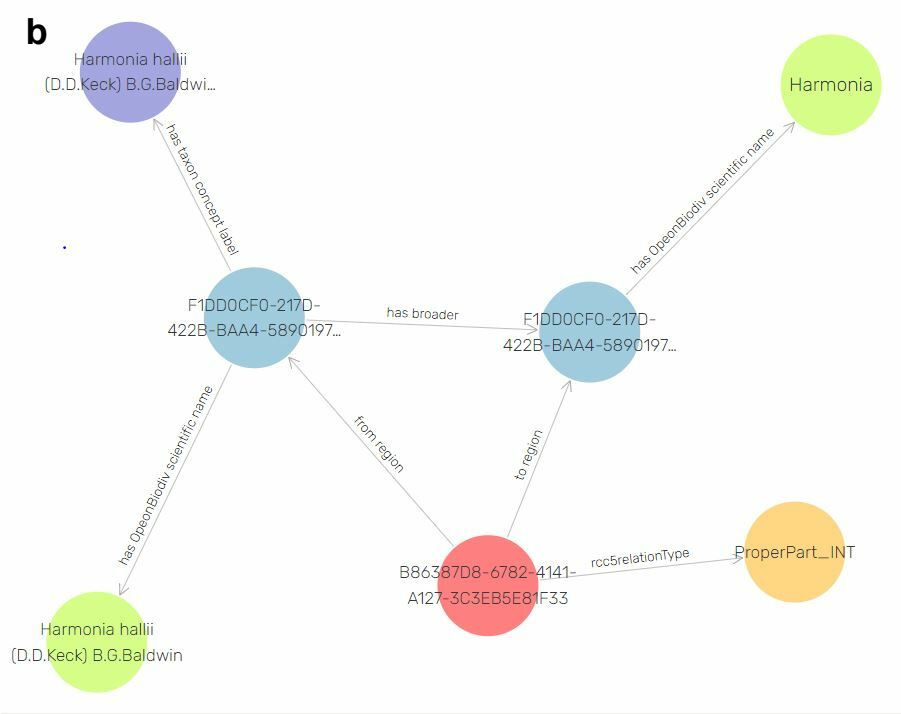
Illustration of the representation of hierarchical information imported from the GBIF Backbone Taxonomy for two taxonomic concepts, Harmonia halii sec. [8] and Harmonia sec. [8]. Each concept has an associated scientific name, denoted via the openbiodiv:hasScientificName property; however, the hierarchical information is not encoded in the names. The hierarchical relationship between Harmonia halii sec. [8] and Harmonia sec. [8] is encoded both via a skos:broader property and reified via the RCC-5 relationship encoded in http://openbiodiv.net/B86387D8-6782-4141-A127-3C3EB5E81F33.

**Figure 4a.**
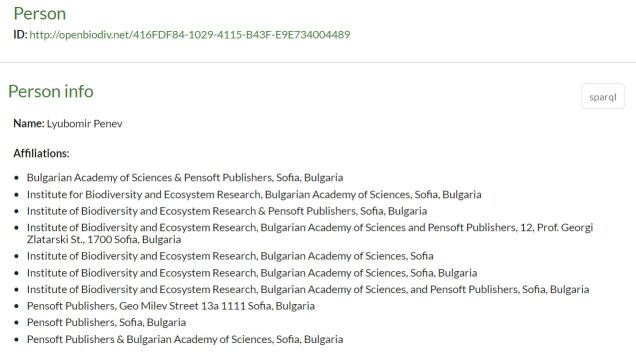


**Figure 4b.**
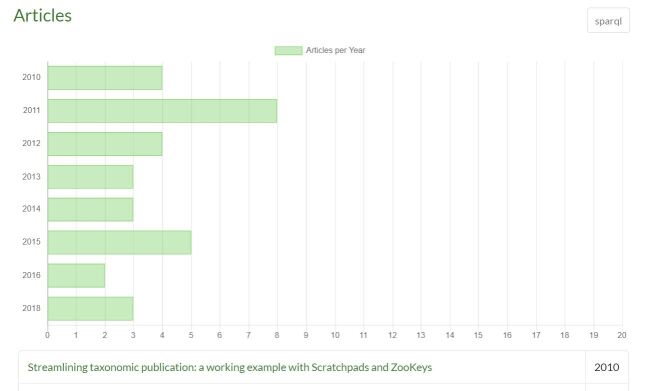


**Table 1.** RDF-ised biodiversity journals published by Pensoft as of 2 March 2021.

**Journal name**	**Number of Articles**	**Number of treatments**
ZooKeys	4715	31966
PhytoKeys	968	4956
Biodiversity Data Journal	695	1360
Journal of Hymenoptera Research	419	1235
Comparative Cytogenetics	338	41
MycoKeys	365	1482
Zoosystematics and Evolution	158	926
Subterranean Biology	152	187
Zoologia	149	78
Nota Lepidopterologica	124	135
Neotropical Biology and Conservation	100	42
Italian Botanist	81	15
Deutsche Entomologische Zeitschrift	80	609
Journal of Orthoptera Research	78	272
Herpetozoa	72	22
African Invertebrates	55	189
Alpine Entomology	54	173
Arctic Environmental Research	50	0
Evolutionary Systematics	41	171
International Journal of Myriapodology	18	97

**Table 2.** Snippet of XML markup of a taxonomic name according to the TaxPub schema and the corresponding RDF triples.

**XML**	<tp:taxon-name> <tp:taxon-name-part taxon-name-part-type = "genus" reg = "Zelus"> P.</tp:taxon-name-part> <tp:taxon-name-part taxon-name-part-type = "species" reg = "casii" >casii</tp:taxon-name-part> </tp:taxon-namе>
**RDF**	http://openbiodiv.net/5BBC353E-CC39-4F2C-B4CE-DC2636CB2DC8 rdf:type openbiodiv:ScientificName; rdfs:label "Zelus casii"; dwc:genus "Zelus"; dwc:specificEpithet "casii"; dwc:verbatimTaxonRank "species"; openbiodiv:hasGbifTaxon openbiodiv:F1DD0CF0-217D-422B-BAA4-58901976D7B4-9146644-scName .

**Table 3.** Data types marked up in articles following TaxPub and TaxonX schemas and the corresponding RDF types of the generated RDF resources. The TaxPub and TaxonX columns contain boolean values (True or False) indicating whether the information about the data type is retrieved from XML files encoded in the corresponding schema or not. For example, Plazi's XMLs, which follow the TaxonX schema, do not contain an Introduction section, hence no resource of type deo:Introduction is created from them.

**Data type**	**TaxPub**	**TaxonX**	**RDF Type**
Article metadata	True	True	fabio:JournalArticle and related
Keyword group	True	False	openbiodiv:KeywordGroup
Abstract	True	True	sro:Abstract
Title	True	True	doco:Title
Author	True	True	foaf:Person
Introduction section	True	False	deo:Introduction
Discussion section	True	True	orb:Discussion
Treatment section	True	True	openbiodiv:Treatment
Nomenclature section	True	True	openbiodiv:NomenclatureSection
Materials examined	True	True	openbiodiv:MaterialsExamined
Diagnosis section	True	True	openbiodiv:DiagnosisSection
Distribution section	True	True	openbiodiv:DistributionSection
Taxonomic key	True	True	openbiodiv:TaxonomicKey
Figure	True	True	doco:Figure
Taxonomic name usage	True	True	openbiodiv:TaxonomicNameUsage
Bibliographic reference list	True	False	doco:BibliographicReferenceList
Bibliographic reference	True	True	deo:BibliographicReference
Institution	True	True	openbiodiv:Institution, openbiodiv:GRSciCollInstitution
Identification	True	True	dwc:Identification
Occurrence	True	True	dwc:Occurrence
Event	True	True	dwc:Event
Location	True	True	dwc:Location

**Table 4.** XML snippet of an author with corresponding RDF

**XML**	<contrib contrib-type ="author" corresp ="no"> <name name-style ="western"> <surname>Zhang</surname > <given names>Guanyang Zhang</given-names> </name> <uri content-type ="orcid">https://orcid.org/0000-0003-4389-4270</uri> <xref ref-type = "aff" rid="3">3</xref> </contrib> <aff id="A3"> <label>3</label> <addr-line>Florida Museum of Natural History, University of Florida, Gainesville, FL, USA</addr-line> </aff>
**RDF**	openbiodiv:51DE6A4F-4651-4540-A54D-21A307105405 rdf:type foaf:Person; rdfs:label "Guanyang Zhang"; foaf:surname "Zhang"; openbiodiv:affiliation "Florida Museum of Natural History, University of Florida, Gainesville, FL, USA"; datacite:hasIdentifier orcid:0000-0003-4389-4270.

**Table 5.** Parent node.

openbiodiv:570F0E79-5632-FF88-A155-73625E50C567 rdf:type fabio:JournalArticle ; prism:doi "10.3897/BDJ.4.e8150" ; dc:publisher "Pensoft Publishers" ; prism:publicationDate "2016-07-08"^^xsd:date ; dcterms:publisher openbiodiv:09EAAD23-3913-421E-9249-3FAAF1BA12DB . openbiodiv:0BD7ED36-1192-47A5-99F9-113998EF3099 rdf:type deo:Introduction ; po:isContainedBy openbiodiv:570F0E79-5632-FF88-A155-73625E50C567 .

**Table 6.** Update rule for replacement name.

PREFIX rdfs: <http://www.w3.org/2000/01/rdf-schema#> PREFIX po: <http://www.essepuntato.it/2008/12/pattern#> PREFIX rdf: <http://www.w3.org/1999/02/22-rdf-syntax-ns#> PREFIX dwc: <http://rs.tdwg.org/dwc/terms/> PREFIX pkm: <http://proton.semanticweb.org/protonkm#> INSERT { GRAPH <http://openbiodiv.net/Updates> { ?name2 openbiodiv:replacementName ?name . } } WHERE { ?tnu1 dwciri:taxonomicStatus openbiodiv:ReplacementName ; pkm:mentions ?name. ?name rdfs:label ?vname ; dwc:verbatimTaxonRank ?rank. ?nomenclature po:contains ?tnu1; po:contains ?citations a openbiodiv:NomenclatureSection. ?citations rdf:type openbiodiv:NomenclatureCitationsList; po:contains ?citation. ?citation po:contains ?tnu2 . ?tnu2 rdf:type openbiodiv:TaxonomicNameUsage ; pkm:mentions ?name2. ?name2 rdfs:label ?vname2. ?name2 dwc:verbatimTaxonRank ?rank. }

**Table 7.** Update rule for related name.

PREFIX rdf: <http://www.w3.org/1999/02/22-rdf-syntax-ns#> PREFIX pkm: <http://proton.semanticweb.org/protonkm#> PREFIX openbiodiv: <http://openbiodiv.net/> PREFIX po: <http://www.essepuntato.it/2008/12/pattern#> PREFIX rdfs: <http://www.w3.org/2000/01/rdf-schema#> INSERT { GRAPH <http://openbiodiv.net/Updates> { ?name2 openbiodiv:relatedName ?name . } } WHERE { ?nom_sec rdf:type openbiodiv:NomenclatureSection ; po:contains ?tnu1 . ?tnu1 rdf:type openbiodiv:TaxonomicNameUsage ; pkm:mentions ?name. ?nom_sec po:contains ?tnu2 . ?tnu2 rdf:type openbiodiv:TaxonomicNameUsage ; pkm:mentions ?name2. FILTER(?name != ?name2) }

**Table 8.** A SPARQL query to retrieve 100 random related taxonomic names

PREFIX openbiodiv: <http://openbiodiv.net/> PREFIX rdfs: <http://www.w3.org/2000/01/rdf-schema#> SELECT * WHERE { ?name_1 openbiodiv:relatedName ?name_2 . ?name_1 rdfs:label ?label_1. ?name_2 rdfs:label ?label_2. } LIMIT 100

**Table 9.** Most profilic author SPARQL query.

PREFIX rdf: <http://www.w3.org/1999/02/22-rdf-syntax-ns#> PREFIX foaf: <http://xmlns.com/foaf/0.1/> PREFIX rdfs: <http://www.w3.org/2000/01/rdf-schema#> PREFIX dcterms: <http://purl.org/dc/terms/> PREFIX fabio: <http://purl.org/spar/fabio/> SELECT (SAMPLE(?name) AS ?name) (COUNT(DISTINCT ?paper) as ?npapers) WHERE { ?author rdf:type foaf:Person ; rdfs:label ?name . ?paper dcterms:creator ?author . ?paper a fabio:ResearchPaper. } GROUP BY ?author ORDER BY DESC (?npapers)

**Table 10.** Most-mentioned scientific name.

PREFIX rdf: <http://www.w3.org/1999/02/22-rdf-syntax-ns#> PREFIX openbiodiv: <http://openbiodiv.net/> PREFIX rdfs: <http://www.w3.org/2000/01/rdf-schema#> PREFIX pkm: <http://proton.semanticweb.org/protonkm#> SELECT (SAMPLE(?name) as ?name) (COUNT(DISTINCT ?tnu) AS ?nmentions) WHERE { ?s rdf:type openbiodiv:ScientificName ; rdfs:label ?name . ?tnu pkm:mentions ?s . } GROUP BY ?s ORDER BY DESC(?nmentions)

**Table 11.** Most-mentioned species name.

PREFIX rdf: <http://www.w3.org/1999/02/22-rdf-syntax-ns#> PREFIX openbiodiv: <http://openbiodiv.net/> PREFIX rdfs: <http://www.w3.org/2000/01/rdf-schema#> PREFIX pkm: <http://proton.semanticweb.org/protonkm#> PREFIX po: <http://www.essepuntato.it/2008/12/pattern#> PREFIX dwc: <http://rs.tdwg.org/dwc/terms/> SELECT ?label (COUNT(?tnu) AS ?nmentions) WHERE { ?s rdf:type openbiodiv:ScientificName ; rdfs:label ?label ; dwc:specificEpithet ?species ; dwc:genus ?genus . ?tnu pkm:mentions ?s . } GROUP BY ?s ?label

**Table 12.** Most-mentioned species name by number of articles that mention it.

PREFIX rdf: <http://www.w3.org/1999/02/22-rdf-syntax-ns> PREFIX openbiodiv: <http://openbiodiv.net/> PREFIX rdfs: <http://www.w3.org/2000/01/rdf-schema#> PREFIX pkm: <http://proton.semanticweb.org/protonkm#> PREFIX po: <http://www.essepuntato.it/2008/12/pattern#> PREFIX fabio: <http://purl.org/spar/fabio/> PREFIX dwc: <http://rs.tdwg.org/dwc/terms/> SELECT (SAMPLE(?name) AS ?n) (COUNT(DISTINCT ?a) AS ?narticles) WHERE { ?s a openbiodiv:ScientificName ; rdfs:label ?name ; dwc:specificEpithet ?sp ; dwc:genus ?g . ?tnu pkm:mentions ?s . ?a po:contains ?tnu ; a fabio:JournalArticle . } GROUP BY ?s ORDER BY DESC(?narticles)

**Table 13.** Most-mentioned scientific names in figure captions.

PREFIX rdf: <http://www.w3.org/1999/02/22-rdf-syntax-ns#> PREFIX openbiodiv: <http://openbiodiv.net/> PREFIX rdfs: <http://www.w3.org/2000/01/rdf-schema#> PREFIX pkm: <http://proton.semanticweb.org/protonkm#> PREFIX po: <http://www.essepuntato.it/2008/12/pattern#> PREFIX doco: <http://purl.org/spar/doco/> SELECT (MAX(?name) AS ?name) (COUNT(DISTINCT ?a) AS ?nmentions) WHERE { ?s rdf:type openbiodiv:ScientificName ; rdfs:label ?name . ?tnu pkm:mentions ?s . ?a po:contains ?tnu . ?a rdf:type doco:Figure . } GROUP BY ?s ORDER BY DESC(?nmentions)

**Table 14.** Figures from a given article.

PREFIX fabio: <http://purl.org/spar/fabio/> PREFIX prism: <http://prismstandard.org/namespaces/basic/2.0/> PREFIX doco: <http://purl.org/spar/doco/> PREFIX c4o: <http://purl.org/spar/c4o/> PREFIX po: <http://www.essepuntato.it/2008/12/pattern#> SELECT ?f WHERE { ?a a fabio:JournalArticle ; prism:doi "10.3897/mycokeys.1.1966" . ?f a doco:Figure . ?a po:contains ?f . }

**Table 15.** Taxonomic discoveries in weevils (Coleoptera, Curculionidae).

PREFIX openbiodiv: <http://openbiodiv.net/> PREFIX dwc: <http://rs.tdwg.org/dwc/terms/> PREFIX rdfs: <http://www.w3.org/2000/01/rdf-schema#> PREFIX dwciri: <http://rs.tdwg.org/dwc/iri/> PREFIX skos: <http://www.w3.org/2004/02/skos/core#> PREFIX prism: <http://prismstandard.org/namespaces/basic/2.0/> PREFIX pkm: <http://proton.semanticweb.org/protonkm#> PREFIX po: <http://www.essepuntato.it/2008/12/pattern#> SELECT * WHERE { ?n rdfs:label "Curculionidae" . ?c openbiodiv:scientificName ?n . ?s skos:broader ?c . ?s openbiodiv:scientificName ?sn . ?sn dwc:genus ?vgenus . ?tnu pkm:mentions ?name; dwciri:taxonomicStatus openbiodiv:TaxonomicDiscovery . ?name dwc:genus ?vgenus; rdfs:label ?verbatim . ?article po:contains+ ?tnu; prism:publicationDate ?date . }

**Table 16.** Sample Lucene query via SPARQL. We have intentionally misspelled the person’s name.

PREFIX inst: <http://www.ontotext.com/connectors/lucene/instance#> PREFIX lucene: <http://www.ontotext.com/connectors/lucene#> PREFIX rdfs: <http://www.w3.org/2000/01/rdf-schema#> SELECT * WHERE { ?search a inst:NewSearch-excluded ; lucene:query "label:Lubomir Penev" ; lucene:entities ?resource . ?resource lucene:score ?score ; rdfs:label ?label . } ORDER BY DESC (?score)

**Table 17.** Asks if the name given by the label has been replaced.

PREFIX rdf: <http://www.w3.org/1999/02/22-rdf-syntax-ns#> PREFIX rdfs: <http://www.w3.org/2000/01/rdf-schema#> PREFIX openbiodiv: <http://openbiodiv.net/> ASK { ?name rdf:type openbiodiv:ScientificName ; rdfs:label "Pentatomidae" . ?name openbiodiv:replacementName ?replacementName . FILTER NOT EXISTS {?replacementName openbiodiv:replacementName ?anotherName .} }

**Table 18.** Asks if the name given by the label is considered unavailable.

PREFIX pkm: <http://proton.semanticweb.org/protonkm#>PREFIX rdfs: <http://www.w3.org/2000/01/rdf-schema#> PREFIX dwciri: <http://rs.tdwg.org/dwc/iri/> PREFIX openbiodiv: <http://openbiodiv.net/> PREFIX prism: <http://prismstandard.org/namespaces/basic/2.0/> PREFIX po: <http://www.essepuntato.it/2008/12/pattern#> ASK { ?tnu pkm:mentions ?name . ?name rdfs:label "Messerschmidia incana G. Mey. 1818" . ?tnu dwciri:taxonomicStatus openbiodiv:UnavailableName . ?article po:contains+ ?tnu . ?article prism:publicationDate ?date . FILTER NOT EXISTS { ?tnu2 pkm:mentions ?name . ?tnu2 dwciri:taxonomicStatus openbiodiv:AvailableName . ?article2 po:contains+ ?tnu2; prism:publicationDate ?date2 . FILTER (?date2 > ?date) } }

**Table 19.** Impact of the fire in Museu Nacional de Rio de Janeiro (MNRJ) on biodiversity knowledge.

PREFIX rdf: <http://www.w3.org/1999/02/22-rdf-syntax-ns#> PREFIX openbiodiv: <http://openbiodiv.net/> PREFIX rdfs: <http://www.w3.org/2000/01/rdf-schema#> PREFIX pkm: <http://proton.semanticweb.org/protonkm#> PREFIX dwc: <http://rs.tdwg.org/dwc/terms/> PREFIX po: <http://www.essepuntato.it/2008/12/pattern#> PREFIX fabio: <http://purl.org/spar/fabio/> PREFIX prism: <http://prismstandard.org/namespaces/basic/2.0/> SELECT ?institution_name (COUNT(?institution_code) AS ?times_mentioned) (COUNT(DISTINCT ?title) AS ?articles) (GROUP_CONCAT(DISTINCT ?title; SEPARATOR=", ") AS ?doi_of_articles) (GROUP_CONCAT(DISTINCT ?name; SEPARATOR=", ") AS ?names_mentioned) (COUNT (DISTINCT ?name) AS ?number_of_taxa) (COUNT(DISTINCT ?tnu) AS ?number_of_tnus) WHERE { BIND("Museu Nacional de Rio de Janeiro (MNRJ)" as ?institution_name) BIND ("MNRJ" as ?institution_code) ?treatment openbiodiv:institutionName|dwc:institutionCode|dwc:collectionCode ?institution_code . OPTIONAL { ?treatment openbiodiv:institutionName ?institution_name } OPTIONAL {?treatment dwc:institutionID <http://grbio.org/cool/zi1i-a0b5>} ?treatment (po:contains)|(po:contains/po:contains) ?tnu; a openbiodiv:Treatment. ?tnu pkm:mentions ?s. ?s a openbiodiv:ScientificName; rdfs:label ?name. ?article po:contains ?treatment ; rdf:type fabio:JournalArticle ; prism:doi ?title . } GROUP BY ?institution_name

**Table 20.** People who have collected specimens belonging to the insect genus *Zelus*.

PREFIX : <http://openbiodiv.net/> PREFIX dcterms: <http://purl.org/dc/terms/> PREFIX frbr: <http://purl.org/vocab/frbr/core#> PREFIX prism: <http://prismstandard.org/namespaces/basic/2.0/> PREFIX dc: <http://purl.org/dc/elements/1.1/> PREFIX fabio: <http://purl.org/spar/fabio/> PREFIX rdf: <http://www.w3.org/1999/02/22-rdf-syntax-ns#> PREFIX po: <http://www.essepuntato.it/2008/12/pattern#> PREFIX openbiodiv: <http://openbiodiv.net/> PREFIX c4o: <http://purl.org/spar/c4o/> PREFIX pkm: <http://proton.semanticweb.org/protonkm#> PREFIX rdfs: <http://www.w3.org/2000/01/rdf-schema#> PREFIX dwc: <http://rs.tdwg.org/dwc/terms/> SELECT ?label ?recorder ?eventDate WHERE { ?article dc:title ?articleTitle ; po:contains ?treatment. ?treatment rdf:type openbiodiv:Treatment; po:contains ?materials; po:contains ?nomenclature. ?materials rdf:type openbiodiv:MaterialsExamined; dwc:occurrenceID ?occurrence; dwc:eventID ?event. ?occurrence dwc:recordedBy ?recorder. ?event dwc:eventDate ?eventDate. ?nomenclature rdf:type openbiodiv:NomenclatureSection; po:contains ?tnu. ?tnu pkm:mentions ?name. ?name rdfs:label ?label; dwc:genus "Zelus". }

**Table 21.** Institutional impact per family.

PREFIX po: <http://www.essepuntato.it/2008/12/pattern#> PREFIX openbiodiv: <http://openbiodiv.net/> PREFIX dwc: <http://rs.tdwg.org/dwc/terms/> PREFIX pkm: <http://proton.semanticweb.org/protonkm#> PREFIX rdfs: <http://www.w3.org/2000/01/rdf-schema#> PREFIX : <http://www.essepuntato.it/2008/12/pattern#> SELECT ?family (COUNT(?treatment) as ?treatment) ?inst ?instName WHERE { ?tnu pkm:mentions ?scName. ?scName dwc:family ?family. ?treatment po:contains ?tnu; a openbiodiv:Treatment; dwc:institutionID ?inst. ?inst a openbiodiv:Institution; openbiodiv:institutionName ?instName. } GROUP BY ?inst ?instName ?family

**Table 22.** Linking holotype descriptions, taxonomy, genomics and institutions.

PREFIX datacite: <http://purl.org/spar/datacite/> PREFIX openbiodiv: <http://openbiodiv.net/> PREFIX deo: <http://purl.org/spar/deo/> PREFIX doco: <http://purl.org/spar/doco/> PREFIX po: <http://www.essepuntato.it/2008/12/pattern#> PREFIX pkm: <http://proton.semanticweb.org/protonkm#> PREFIX rdfs: <http://www.w3.org/2000/01/rdf-schema#> PREFIX dwc: <http://rs.tdwg.org/dwc/terms/> PREFIX fabio: <http://purl.org/spar/fabio/> PREFIX rdf: <http://www.w3.org/1999/02/22-rdf-syntax-ns#> PREFIX prism: <http://prismstandard.org/namespaces/basic/2.0/> SELECT ?materialsExamined ?genomicLabel ?system?name ?label ?institution ?doi WHERE { ?genomicIdentifier datacite:usesIdentifierScheme ?system; rdfs:label ?genomicLabel. FILTER (?system IN (datacite:genbank, datacite:boldsystems)) . ?materialsExamined openbiodiv:mentionsIdentifier ?genomicIdentifier; a openbiodiv:MaterialsExamined; po:contains ?holotypeDescr. ?holotypeDescr a openbiodiv:HolotypeDescription. ?treatment po:contains ?materialsExamined; a openbiodiv:Treatment; po:contains ?nomenclature; dwc:institutionID ?institution. ?nomenclature a openbiodiv:NomenclatureSection; po:contains ?tnu. ?tnu pkm:mentions ?name. ?name rdfs:label ?label. ?article a fabio:JournalArticle; po:contains ?treatment; prism:doi ?doi. }

## Footnotes

*1
https://github.com/pensoft/OpenBiodiv/blob/master/ontology/openbiodiv-ontology-mod.ttl

*2
https://github.com/pensoft/openbiodiv-o/tree/master/imports

*3
https://github.com/pensoft/ropenbio/blob/master/R/taxpub.R

*4
https://github.com/pensoft/ropenbio/blob/master/R/taxonx.R
